# UNC-Utah NA-MIC framework for DTI fiber tract analysis

**DOI:** 10.3389/fninf.2013.00051

**Published:** 2014-01-09

**Authors:** Audrey R. Verde, Francois Budin, Jean-Baptiste Berger, Aditya Gupta, Mahshid Farzinfar, Adrien Kaiser, Mihye Ahn, Hans Johnson, Joy Matsui, Heather C. Hazlett, Anuja Sharma, Casey Goodlett, Yundi Shi, Sylvain Gouttard, Clement Vachet, Joseph Piven, Hongtu Zhu, Guido Gerig, Martin Styner

**Affiliations:** ^1^Neuro Image Research and Analysis Laboratory, Department of Psychiatry, University of North CarolinaChapel Hill, NC, USA; ^2^Children’s Hospital of Pittsburgh, University of PittsburghPittsburgh, PA, USA; ^3^Department of Biostatistics, University of North CarolinaChapel Hill, NC, USA; ^4^Iowa Institute for Biomedical Imaging, University of IowaIowa City, IA, USA; ^5^Scientific Computing and Imaging Institute, University of UtahSalt Lake City, UT, USA; ^6^Kitware Inc.Clifton Park, NY, USA; ^7^Department of Computer Science, University of North CarolinaChapel Hill, NC, USA

**Keywords:** neonatal neuroimaging, white matter pathways, magnetic resonance imaging, diffusion tensor imaging, diffusion imaging quality control, DTI atlas building

## Abstract

Diffusion tensor imaging has become an important modality in the field of neuroimaging to capture changes in micro-organization and to assess white matter integrity or development. While there exists a number of tractography toolsets, these usually lack tools for preprocessing or to analyze diffusion properties along the fiber tracts. Currently, the field is in critical need of a coherent end-to-end toolset for performing an along-fiber tract analysis, accessible to non-technical neuroimaging researchers. The UNC-Utah NA-MIC DTI framework represents a coherent, open source, end-to-end toolset for atlas fiber tract based DTI analysis encompassing DICOM data conversion, quality control, atlas building, fiber tractography, fiber parameterization, and statistical analysis of diffusion properties. Most steps utilize graphical user interfaces (GUI) to simplify interaction and provide an extensive DTI analysis framework for non-technical researchers/investigators. We illustrate the use of our framework on a small sample, cross sectional neuroimaging study of eight healthy 1-year-old children from the Infant Brain Imaging Study (IBIS) Network. In this limited test study, we illustrate the power of our method by quantifying the diffusion properties at 1 year of age on the genu and splenium fiber tracts.

## INTRODUCTION

Since its invention in the 1980s diffusion weighted imaging has become increasingly popular for the analysis of brain pathologies and development. It is now standard practice for investigators to collect diffusion weighted images (DWI) concurrently with other modalities of interest without any expertise on how to preprocess or analyze DWI. To address this growing wealth of DWI data, we have created a coherent framework of tools for the pre-processing and analysis of DWI and diffusion tensor images (DTI) that is accessible for the non-technical user. It is the aim of this paper to describe our DTI processing workflow and to provide example data processed with this framework for increased workflow clarity.

Diffusion weighted images capture the movement of water molecules. When locally unconstrained, such water molecules move by Brownian motion in an isotropic spherical fashion, for example as is the case within the brain lateral ventricles. However when locally confined, the diffusion of water molecules is constrained by local boundaries, such as cell membranes. Within the human brain white matter (WM), largely composed of neuronal axons, the diffusion of water is more ellipsoid or anisotropic in shape due to the confines of the internal microstructure and the myelin surrounding neuron axons.

Characterizing the properties of diffusion can inform us about the integrity of WM micro-structure and provides insight on the mechanisms and progression of disease. To date DTI has been used in research to further the understanding of pathology in many neurologic diseases including multiple sclerosis (Zhu et al., 2013), Alzheimer’s (Patil et al., 2013), and Parkinson’s (Du et al., 2012), as well as normal neurodevelopment (Geng et al., 2012), and aging (Lebel et al., 2012). The most common properties measured in DWI/DTI are axial (AD, λ_∣ ∣_), radial (RD, λ_⊥_), and mean diffusivity (MD), as well as fractional anisotropy (FA). Axial diffusivity has been shown to decrease in the case of cell death, while radial diffusivity will increase in the case of myelin damage (Song et al., 2003). Alterations in FA reflect changes of AD, RD, and MD and describe the overall pattern of diffusion.

Several analysis frameworks exist for the study of DWI/DTI data, typically falling into one of three categories: region of interest (ROI) based analysis, voxel based analysis (VBA), and quantitative tractography. ROI driven analysis can be performed by registering individual subject DWI images either to their corresponding anatomical images or by registering the DWI of all individuals to a prior existing atlas. In the first type of ROI analysis, regional segmentations from structural (T1w, T2w) MRIs of the same subject are propagated via linear or deformable co-registration of the structural images to the diffusion image data and mean statistics are collected (Shi et al., 2013). However, the structural WM parcellations are commonly lobar based and thus cannot separate the measurements from different fiber tracts located in the same cortical lobe/region. In the second type of ROI analysis co-registration with a prior WM fiber atlas is performed (Faria et al., 2010). This type of analysis again requires deformable registration and results in a single measure per fiber region, potentially combining regions that show significantly different fiber situations. The positive aspects of both of these types of region based analysis is that processing is relatively simple and it is robust against imperfect registration. On the other hand, the analysis of large ROIs means non-specific findings as ROIs can contain multiple fiber tracts and multiple fiber situations (single, fanning, crossing fiber situations). This form of analysis thus leads to limited localization of findings.

Another DTI processing framework is VBA. This style of analysis requires registration of each subject into a study specific reference space and then all voxels are tested individually for significance (Liu et al., 2009). While this technique allows for whole brain analysis and is suitable for hypothesis generation, this analysis assumes absolutely perfect registration on a voxel by voxel basis and is thus highly sensitive to the deformable co-registration. Another form of VBA is FSL’s Tract Based Spatial Statistics (TBSS). Here a WM skeleton is created for the brain, maximal FA values are projected to the WM skeleton, and voxel wise analysis of the skeleton is performed (Smith et al., 2006). Due to the projection to the WM skeleton, TBSS is less sensitive to registration accuracy compared to standard VBA. While FSL’s TBSS tool provides a coherent framework that works well with imperfect registration, it does not have an explicit tract representation and instead provides a skeletal voxel representation that cannot be uniquely linked to individual fibers throughout the brain. Further, the use of maximal FA values renders TBSS sensitive to DWI artifacts.

Our framework falls into the category of quantitative tractography, where anatomically informed curvilinear regions are used to analyze diffusion at specific locations all along fiber tracts. Diffusion property profiles are computed as weighted averages taking into account the neighborhood at each step along the fiber bundles (Goodlett et al., 2009). This form of analysis results in highly localized statistics that can be visualized back on the individual fiber bundles.

In this paper, we propose the use of our 3D Slicer based UNC-Utah NA-MIC DTI framework for a coherent atlas fiber tract based DTI analysis. Most steps of the framework utilize graphical user interfaces (GUI) to simplify interaction and provide accessibility for non-technical researchers. At the heart of the framework is a set of tools anchored around the multi-purpose image analysis platform 3D-Slicer. Several workflow steps are handled via external modules called from Slicer in order to provide an integrated approach. Our workflow starts with data conversion from DICOM format, followed by an automatic as well as interactive DWI and DTI quality control (QC) steps. Though underappreciated in the field, appropriate and thorough QC is a must for every DTI study. Our framework then centers around a DTI atlas that is either provided as a prior template or computed directly as an unbiased average atlas from the study data via deformable atlas building (Goodlett et al., 2009). Fiber tracts are defined via interactive label map tractography on that atlas followed by consecutive interactive fiber cleaning steps. Individual subject DTI fiber tract profiles of FA, MD, RD, and AD are extracted automatically using the atlas mapping information. These fiber tract profiles are then analyzed using our statistics toolbox (FADTTS; Zhu et al., 2011). The local statistical results are then mapped back on to the fiber bundles for visualization within 3D Slicer (Pieper et al., 2004).

This framework has been developed over several years and we continuously improve the current toolset. Within the past year we have added entropy-based detection of vibration artifacts as part of the automatic QC and an interactive atlas building tool called DTIAtlasBuilder. All tools that are part of the UNC-Utah NA-MIC DTI framework are open source and available on NITRC, providing the neuroimaging field with a transparent and coherent toolset for DTI fiber tract based analysis.

We illustrate the use of our framework on a small sample, cross sectional neuroimaging study of eight healthy 1-year-old children from the Infant Brain Imaging Study (IBIS) Network (). In this paper, we demonstrate our method by quantifying the diffusion properties within the genu and splenium fiber tracts. The corresponding raw data, intermediate files, and final results for data presented in this manuscript have been made freely available on NITRC.

## MATERIALS AND METHODS

**FIGURE 1:**
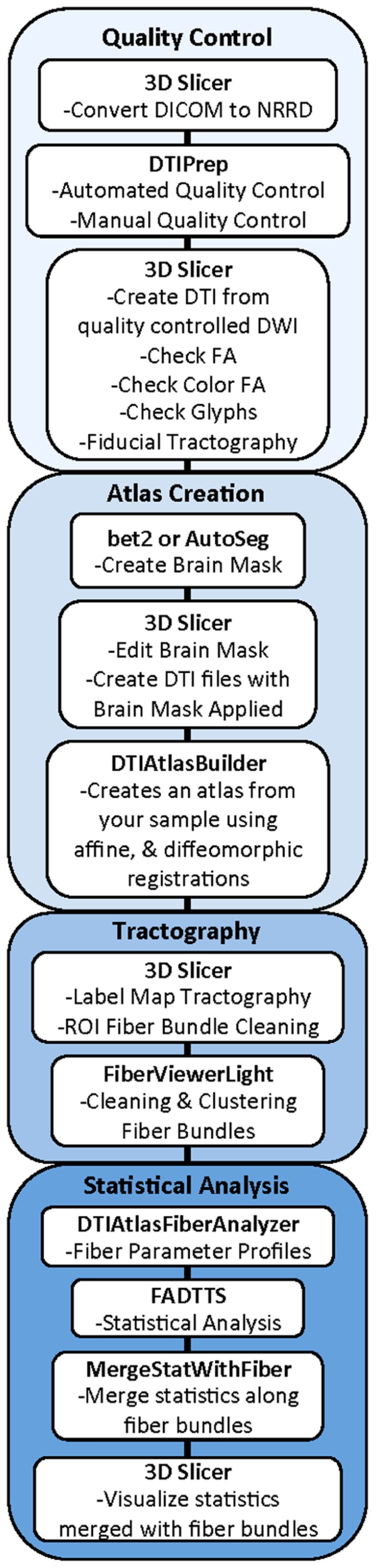
**UNC-Utah NA-MIC DTI framework.** Step 1 is quality control. Step 2 is atlas creation. Step 3 is interactive tractography. Step 4 is parameter profile creation and statistical analysis.

**FIGURE 2:**
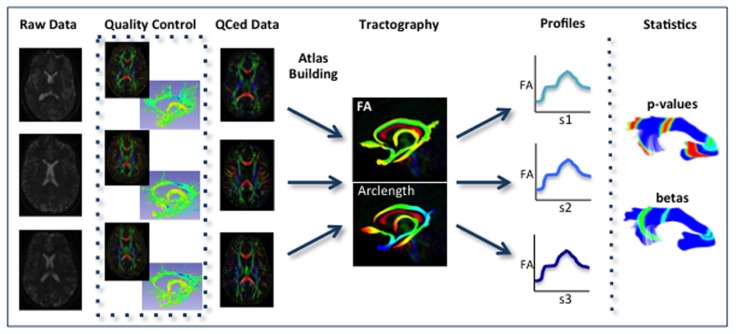
**Schematic of DTI processing workflow.** The input raw DWI data first undergoes vigorous quality control before the final conversion to DTI for Atlas building. Interactive tractography is performed on the Atlas. From these fiber bundles, diffusion parameter profiles are extracted and analyzed along with other study variables to determine significant interactions.

Our framework for the fiber tract based analysis of DTI images is composed of four essential segments: (1) QC, (2) Atlas Creation, (3) Interactive Tractography, and (4) Statistical Analysis. The tool workflow can be seen in **Figure 1** and an overall conceptual schematic of the process is displayed in **Figure 2**. All of the tools referenced in the description of our workflow can be utilized as stand-alone command line applications to facilitate scripting and grid computing, or interactively as part of 3D Slicer as external modules. While the first segment of our framework is termed QC, it is critical to perform quality assessment of the processing at every step in the framework to ensure the data that is analyzed is correct.

### DWI AND DTI QUALITY CONTROL

#### DWI quality control – DTIPrep

**FIGURE 3:**
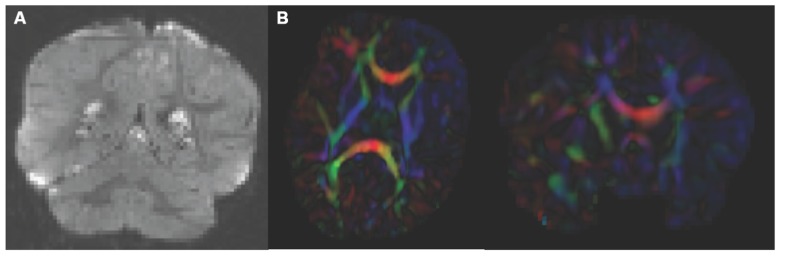
**Artifacts encountered during quality control. (A)** DWI artifact missed by automatic quality control run of DTIPrep, but caught in the visual quality control. You can see the horizontal lines running through this coronal view of the brain. Toward the top, almost an entire slice is missing. In cases like this, the gradient containing this artifact would be excluded from the saved DWI volume. **(B)** Axial and coronal view of DTI color fractional anisotropy (FA) volume. The large area of blue in the left frontal and temporal lobes indicates that a dominant direction artifact remains after DTIPrep quality control. Thus this artifact was too large to correct and this participant was excluded from analysis.

Analysis starts by converting the raw DWI from DICOM to NRRD format using a tool called DicomToNrrdConverter in 3D Slicer. This tool is capable of computing DWI via the b-matrix information stored in Siemens DICOM headers, as well as recognizing many other manufacturer specific tags. A tool called DTIPrep (Liu et al., 2010) is then used to check for and correct several common artifacts found in DWI. In summary, DTIPrep performs image information checking, slice-wise artifact detection, creates a baseline average, corrects for subject motion and eddy current induced deformations, and finally detects dominant direction artifacts (Farzinfar et al., 2013). To carry out the automatic QC a protocol must be created that contains all parameters of the DTIPrep QC. Protocols are fully modifiable for individual studies and include a significant number of parameters for every step. DTIPrep gives the option of creating a default protocol from a given scan. On completion of the automatic QC it is further advised to perform additional visual QC. Here artifacts that may be missed by the automatic QC step can be detected and gradients still containing artifacts or motion can be excluded from the final DWI (**Figure 3A**). After visual QC, the DTI tensor and property images are then automatically estimated from the DWI data via the dtiestim and dtiprocess modules.

#### DTI quality control – 3D slicer

In addition to checking the DWI’s for artifacts, the resulting DTI must also undergo visual QC using 3D Slicer. This QC step is to ensure the correct directional encoding, sufficient signal-to-noise (SNR) in the FA data, and to test the overall quality of the data by performing landmark-based/“fiducial” tractography.

Visual DTI QC begins by loading the FA image and scrolling through the slices for each plane (axial, sagittal, coronal) to assess the apparent level of SNR for the volume. Next, the DTI volume is analyzed to determine if the header information encoding directionality for the volume is correct. The DTI image is colored by tract direction with tracts moving right to left colored red, tracts moving anterior to posterior colored green, and tracts moving inferior to superior colored blue. The corpus callosum (CC) should appear red, the cingulum green, and the corticospinal tract should be blue. Also special attention is directed to detect dominant directional artifacts if low FA regions (such as the gray matter) display a single color throughout most of the DTI image, as seen in **Figure 3B**. Visualization of the primary direction via line glyphs is the next step to ensure the correctness of the diffusion measurement frame. Glyphs should follow the direction of the main fiber tracts. If the glyphs are incorrect (due to incorrect DICOM information or scanner software malfunction), then the corrected measurement frame must be determined and edited in the volume header.

Once the header information is confirmed to be correct for the volume, fiducial based tractography will provide information whether tractography is feasible, and anatomically accurate. Fiducial tractography is commonly performed for the major fiber tracts (CC genu and splenium, cingulum, and corticospinal tract) as well as any tracts of particular interest for the neuroimaging study at hand. In the resulting fiber tracts, it is important to observe (a) sufficient quantity of fibers delineating a fiber tract, (b) whether fibers completely track between the expected terminal regions, and whether the fiber location is anatomically accurate throughout the length of the fiber tract. If artifacts remain in the volume, then fibers may break prematurely, appear warped, or incorrectly localized, or if the data has a very low SNR the fiber bundles of interest may be impossible to track. If tracking is not feasible or anatomically incorrect, then exclusion of this dataset is suggested.

### ATLAS BUILDING

#### Brain mask creation, editing, and application – bet2/AutoSeg

The first step in atlas building is to extract out the brain regions for each DTI image. To do this, a brain mask must be computed, edited, and applied to the DWIs in order to create a skull-stripped DTI. Many tools are available for brain mask creation, such as the “Brain Mask from DWI” module in 3D Slicer, bet2 within DTIPrep, or a tissue segmentation based approach in AutoSeg (Van Ginneken et al., 2007). Brain mask creation by bet2 can be computed using the baseline image or the IDWI (isotropic DWI), while both are used jointly to compute the brain mask with AutoSeg. Mask editing is performed also within 3D Slicer, but any editing tools with Slicer compatible format (NRRD, Nifti, GIPL, Analyze) can be employed such as itk-SNAP (Yushkevich et al., 2006). The edited brain mask can then be applied to the DWIs, creating a skull stripped DTI (referred to as “Original DTI” in this paper) ready for atlas building.

#### Atlas creation – DTIAtlasBuilder

**FIGURE 4:**
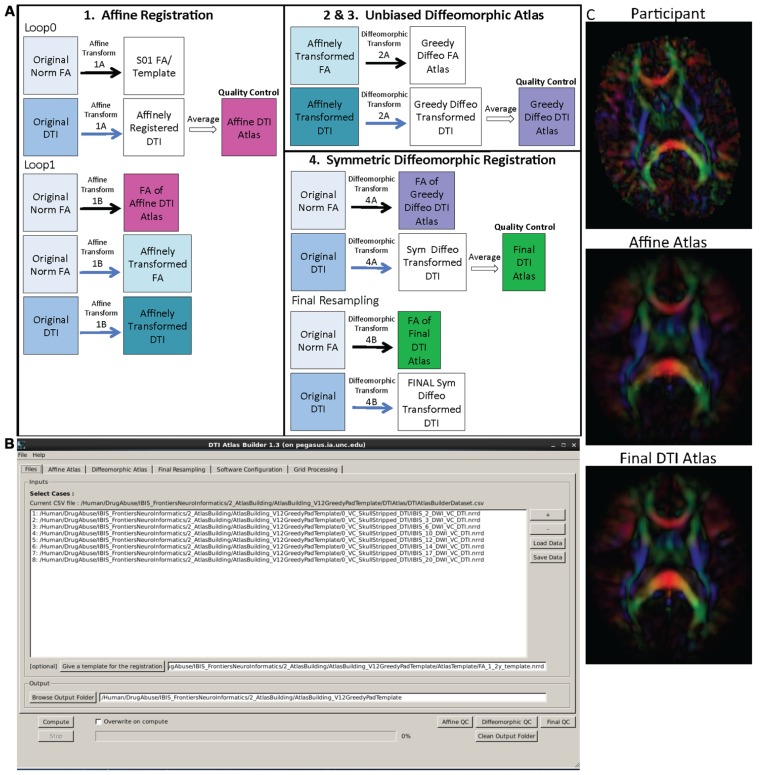
**DTIAtlasBuilder steps, GUI, and registration progression. (A)** DTIAtlasBuilder steps. Black solid arrows indicate transform computation. Blue solid arrows indicate transform application. Hollow black arrows indicate an averaging of transformed images. Before the Affine registration in step 1, fractional anisotropy (FA) is normalized so that FA intensity difference between subject images does not bias the atlas creation. The FA is also “filtered” to remove negative eigen values, which will adjust the resulting FA values to scale between zero and one. The transform computed in step 4 final resampling, diffeomorphic transform **(B)**, is termed the Final (or Global) Displacement Field. It is this transform that is required to extract fiber tract profiles from each individual subject DTI when using DTIAtlasFiberAnalyzer later in the workflow. **(B)** DTIAtlasBuilder GUI. Here one can see the first tab with the input list of quality controlled and skull-stripped DTI volumes. Along the top of the GUI, the tabs for adjusting parameters and tool paths are visible. **(C)** Progression of the atlas building from a single participant volume to the affine atlas, and the final unbiased diffeomorphically registered DTI Atlas in the presented experimental pediatric population.

The main atlas building step is performed with a tool called DTIAtlasBuilder, detailed in **Figure 4**. This program allows the user to create an atlas image as an unbiased average of all co-registered DTI images in the study. Atlases are created iteratively starting from affine atlases (step 1) and moving to deformable diffeomorphic atlases (step 2, 3, and 4). The registration is done in three steps: (1) affine registration with the Slicer General Registration module (called BRAINSFit; Johnson et al., 2007), (2) unbiased diffeomorphic atlas computation (Joshi et al., 2004) with the GreedyAtlas module within AtlasWerks (Joshi et al., 2004), and (3) a refinement step via symmetric diffeomorphic registration with the Slicer DTI registration module DTI-Reg using the Advanced Normalization Tools (ANTS; Avants et al., 2008; Klein et al., 2009). The final, refinement step significantly improves registration accuracy in our experience, most likely attributable to the use of local normalized cross-correlation as the image similarity metric. A final step will combine multiple transforms to compute the overall transformation (GlobalDisplacementFields) from the original DTI images to the Final DTI Atlas. This final transform is required for the analysis of fiber tract properties in the individual subject space.

In the first step, DTIAtlas Builder will generate scalar FA images from the skull stripped DTIs. The affine atlas building starts by registering subject images to a pre-exiting FA template in MNI-normative space or to the first subject in case no prior template is specified. In order to avoid biasing the subsequent analysis, the image intensities are calibrated with the template’s intensities using standard histogram quantile normalization available in ITK. The affine transforms are applied to the original DTIs and the transformed images are averaged to create the Affine DTI Atlas (see **Figure 4**). Each image’s affine transform is then updated by recomputing the affine registration in a second loop to the Affine DTI Atlas (rather than to the initial template as in the first loop). This transform, Affine Transform 1B, is then applied to the original DTI to create affinely co-registered DTI’s for each subject.

In the second step of atlas building, the unbiased diffeomorphic atlas is computed from the affinely registered FAs via diffeomorphic, fluid flow based atlas building. This results in deformation fields from the affine space to the unbiased atlas space. These deformation fields are then applied to the affinely registered DTIs and the transformed images are averaged to create a Greedy Diffeomorphic DTI Atlas (**Figure 4**).

The last stage of atlas building utilizes DTI-Reg to refine the diffeomorphic registration computed in the previous step. To start, FA images from the original subject DTIs are histogram normalized to the Greedy Diffeomorphic FA Atlas, and then diffeomorphically registered to it. The resulting deformation fields are then applied to the original subject DTI and the resulting diffeomorphically registered DTIs are averaged to create the Final DTI Atlas. The deformation fields are then updated with another loop of registrations with DTI-Reg in order to co-register the images with the Final DTI Atlas (rather than with the Greedy Diffeomorphic FA Atlas as in the first loop). The overall Global Displacement Fields are finally computed by composing the affine transform from step 1, as well as the affine and deformation transform from this last step. These displacement fields will be used to map the fiber tracts created in atlas space to the individual subject spaces for analysis. The final atlas co-registered DTI images are computed by applying these final deformations fields. It is noteworthy that this deformation of the DTI images is unlike the processing in TBSS/FSL, where FA images are deformed. Furthermore, all DTI image interpolations are performed using standard Log Euclidean based resampling (Arsigny et al., 2006).

With the atlas building complete, it is important to perform QC on the Affine, Greedy Diffeomorphic, and Final DTI Atlases, simply accessible with a single button within DTIAtlasBuilder. In this QC step we can visually determine the point to point correspondence between the subject images and the atlas to determine if computed registration transforms are appropriate. The progression of atlas sharpness can be seen in **Figure 4C**.

Software path configuration for the tool can be performed automatically or manually, and the settings can be saved for future use. This tool also has a “no GUI”/command line option, given that the data file, parameter file, and software configuration file are available (all can be determined and saved via the GUI). There is also direct support for the GRID software LSF (Load Sharing Facility, Platform Computing, Inc).

### TRACTOGRAPHY

#### Label map single tensor tractography – 3D slicer

**FIGURE 5:**
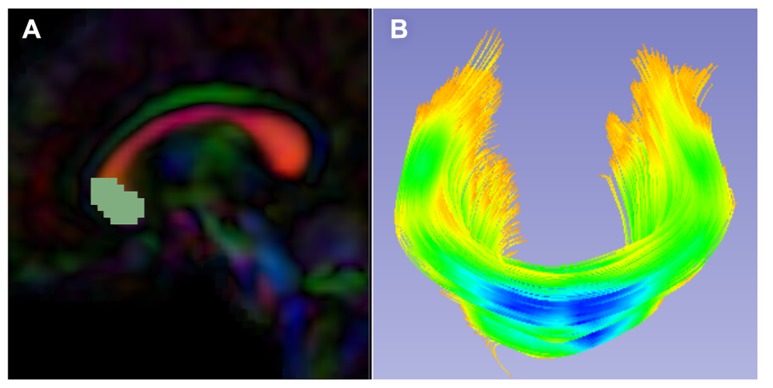
**Slicer label map tractography. (A)** Sagittal view of the corpus callosum genu label map in the Final DTI Atlas. **(B)** Resulting genu bundle fibers from label map tractography within 3D Slicer. The bundle is colored by FA values along the fibers with cooler colors indicating higher FA.

While atlas building is fully automatic, the definition of fiber tracts of interest needs to be performed interactively. Fiber tractography only needs to be performed once on the Final DTI Atlas built in the prior step. In our framework, fiber tractography is performed via the label map tractography module or the full brain tractography module within 3D Slicer. If using label map tractography, each fiber tract has a separate anatomically informed label created in the editor module and is tracked individually (**Figure 5**). Alternative fiber tracking tools can be employed as long as the resulting fibers are stored in standardized vtk-based file format. Resulting DTI fiber bundles require post-processing via ROIs to remove unwanted, erroneous fibers.

#### Coarse fiber bundle editing with ROIs – 3D slicer

The results of label map tractography may contain fibers in addition to the fiber bundle of interest. Coarse cleaning is performed with interactive 3D regions of interest (ROIs) in 3D Slicer’s tractography display module. Here fibers to be kept or removed are selected using ROIs iteratively. ROIs should also be utilized for separating tracts that run within the left and right hemispheres in order to allow separate analysis of the right and left portions (for example the left and right hemispheric portions of the fornix).

#### Fine fiber bundle editing – FiberViewerLight

**FIGURE 6:**
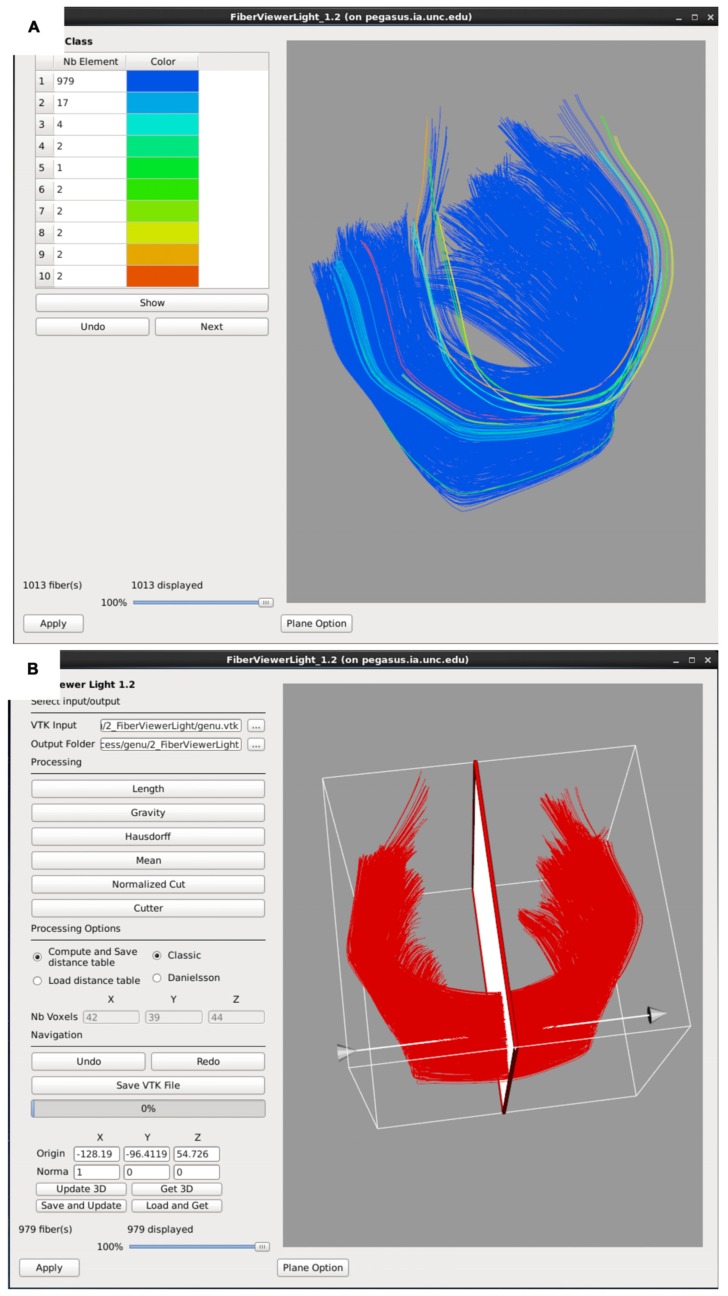
**FiberViewerLight clustering, cleaning, and plane creation. (A)** Example of genu fiber clustering based on the center of gravity. **(B)** In the left panel all of the fiber clustering algorithms are visible, as well as the different styles of computation (Classic vs Danielsson). In the right panel the plane function is visualized for the genu. This tool is used to set a plane of origin from which the fiber bundle will be parameterized for further analysis.

Fiber tracts often need additional, finer editing via clustering and cleaning within FiberViewerLight. Fiber length based cleaning and several fiber clustering algorithms (center of gravity, Hausdorff distance, mean distance, normalized cut) to further delineate which fibers belong in the bundle of interest and which ones do not (**Figure 6**). The decision for inclusion or exclusion is based on expected fiber tract anatomy. Once fiber bundles are deemed appropriate, diffusion property profiles for each fiber tract and subject image are extracted as described in the next section.

### PROPERTY PROFILES AND STATISTICAL ANALYSIS

#### Diffusion property profiles – DTIAtlasFiberAnalyzer

As a final step prior to statistical analysis, diffusion properties along the fiber tracts, called fiber property profiles, are extracted. For that purpose, a fiber tract parameterization needs to be established, which is achieved via arclength parameterization (Corouge et al., 2005) starting from each fiber’s intersection with an “origin” plane. While DTIAtlasFiberAnalyzer automatically determines an appropriate origin plane, investigators can choose to interactively determine that plane in order to associate a specific anatomical fiber location to this origin within FiberViewerLight (see **Figure 6B**).

**FIGURE 7:**
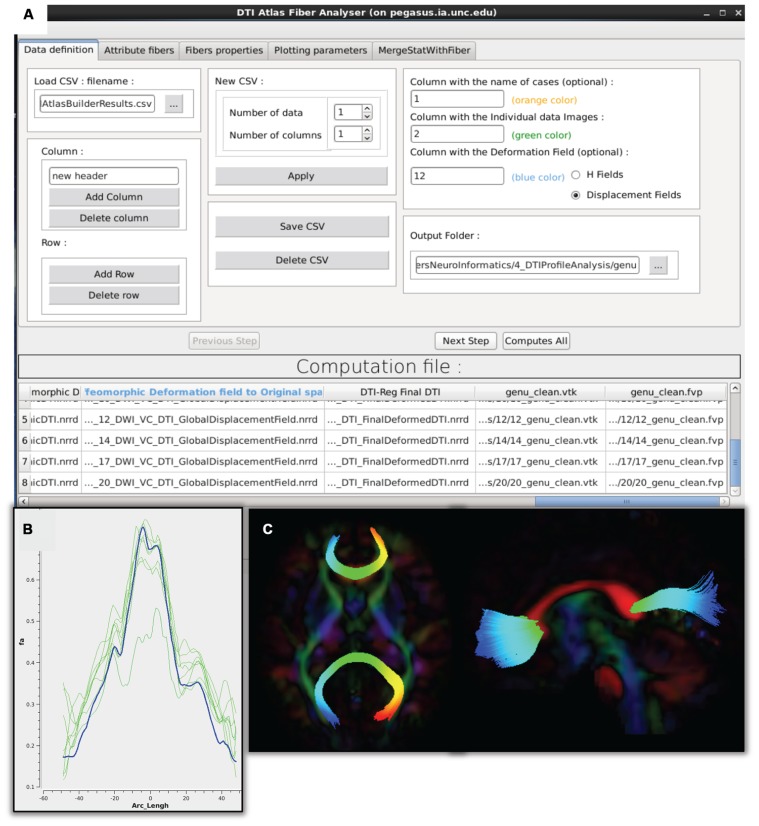
**DTIAtlasFiberAnalyzer GUI. (A)** In the first tab of this tool the input data is defined: the label for each participant, the original quality controlled skull-stripped DTI, and the GlobalDeformationField computed in Atlas Building. **(B)** After DTIAtlasFiberAnalyzer has sampled the intended diffusion parameters, DTI property profiles for individual images (green) and the DTI atlas (blue) can be visualized in the Plot Parameters tab. Inspecting these profiles allows for a different type of quality control of the atlas registrations. The FA profiles of the subjects (pictured here) should be similarly shaped to the atlas. **(C)** Visualization of the parameterized genu and splenium fibers in the axial and sagittal views. Fibers that have been correctly parameterized with DTIAtlasFIberAnalyzer via the origin plane will be colored by FiberLocationIndex (arclength) from red to blue. Red indicates low arclength and blue high arclengths. The corresponding profile plots of these genu and splenium fibers would run from the right to the left hemisphere.

The parameter profile generation and processing is performed with a tool called DTIAtlasFiberAnalyzer, shown in **Figure 7**. The profile extraction operates on the original DTI data in order to avoid the need for tensor deformation by deforming the parameterized fibers into original space via the previously computed Global Deformation Field. At the deformed fiber locations the tool can then calculate the different fiber property profiles along each fiber in the bundle (FA, RD, AD, MD, GA, Fro, lambda 2, and lambda 3) in subject space. **Figure 7B** shows example FA profiles of the genu calculated and visualized with DTIAtlasFiberAnalyzer for this study. Visualization of these fiber property profiles for each subject versus the fiber bundle in atlas space allows an alternative assessment of the registration quality to atlas space (**Figure 6B**). Inspecting the atlas fiber bundle parameterization is an additional QC step to confirm correct parameterization (**Figure 7C**). An error in parameterization usually requires a change in origin plane to correct the problem.

**FIGURE 8:**
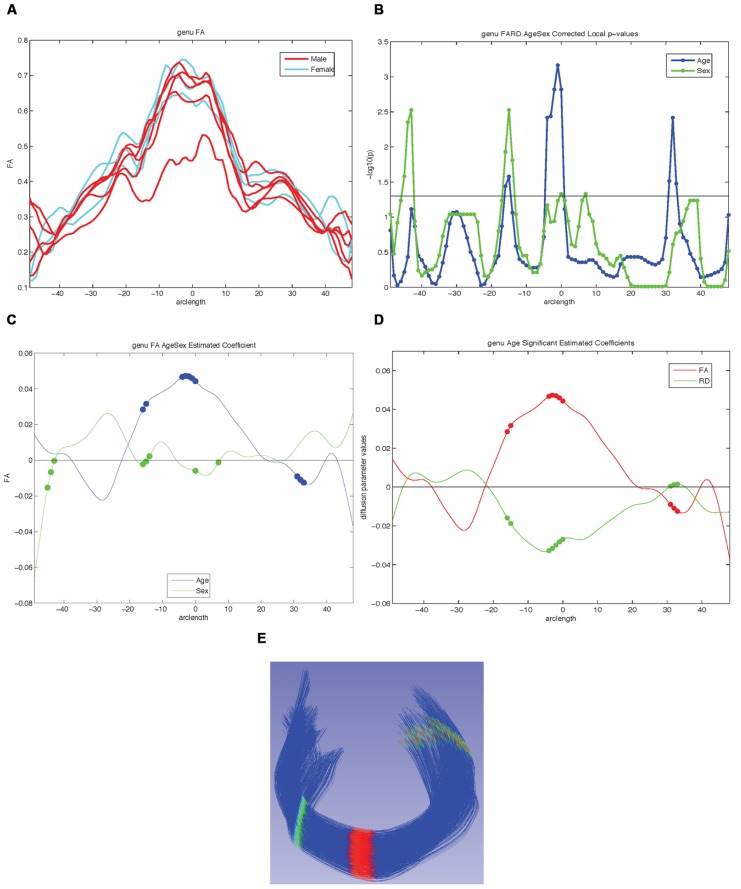
**Examples of possible plots from functional analysis of diffusion tensor tract statistics (FADTTS) and MergeStatWithFiber. (A)** Plot the raw data. **(B)** Output of FDR corrected local-log *p*-values along the length of the fiber tract. **(C)** Separate beta plots for all of the covariates entered into the model and how they interact with each diffusion parameter. **(D)** Separate beta plots for how all the investigated diffusion parameters interact with each covariate in the model. For both beta plots, the filled in circles along the curves indicate areas of significance post FDR correction. **(E)** Visualization of local statistics along the tract with 3D Slicer. Specifically this image reflects the corrected local *p*-values for the interaction between Age and FA for the genu [**(C)** blue line, **(D)** red line]. All non-significant points are assigned a single value and color (dark blue here). Points that are significant then proceed from the next value on the color bar until the furthest end of the color bar. In the color bar shown, areas of significance are colored from cyan to red, with red areas indicating most significance. Any color bar available can be used.

The fiber property profiles are the input for the analysis tool called Functional Analysis of Diffusion Tensor Tract Statistics (FADTTS; Zhu et al., 2011) that performs statistical analysis on the fiber bundle profiles. This tool has the ability to compute group difference and correlational analyses (**Figure 8**). Currently the FADTTS tool is Matlab (MathWorks Inc, MA, USA) based, and requires a user familiar to Matlab to operate. The Matlab scripts used to analyze the data for this paper are available with the sample data at 

#### Statistical analysis of diffusion properties along the fiber tract – FADTTS

Using the diffusion profiles obtained from DTIAtlasFiberAnalyzer, it is possible to test how these diffusion properties relate to other variables of interest within the sample data. FADTTS performs several statistics tests including a multi-variate coefficient model, weighted least squares estimation, and functional principal component analysis. These tests produce both global and local test statistics with confidence bands and the local values are corrected for multiple comparisons with false discovery rate (FDR). Several statistical plots are commonly created, a sample of which can be seen in **Figure 8**. From the beta plots, the direction, magnitude, and significance of the relationship of interest can be assessed by the beta sign, distance from zero, and the significance marker (a filled circle) on the curve, respectively (**Figures 8C,D**).

#### Mapping covariate p-values on fiber bundles for visualization – MergeStatWithFiber

Upon computation of test statistics with FADTTS, the statistics can be mapped to their corresponding location on the fiber bundle using a tool called MergeStatWithFiber. The input for MergeStatWithFiber is the comma-separate-value (csv) file resulting from FADTTS with the addition of arclength as the first column, and a header row containing column labels. The result of this merge is then visualized in 3D Slicer (**Figure 8E**). The color map shown is determined by the significance threshold set in MergeStatWithFiber, as such that all points on the fiber that have a *p*-value above the significance threshold are colored blue, and all points with values below the significance threshold are colored from cyan to red, with points indicated in red signifying the areas of greatest significance. If desired, this color map can be altered within Slicer to another color table. MergeStatWithFiber can be used as a command line tool or through the final tab in the DTIAtalsFiberAnalyzer GUI (**Figure 7**).

## RESULTS

For our example data we created an unbiased diffeomorphic atlas with eight 12-month-old IBIS participant scans, and performed interactive tractography of the genu and splenium on this atlas. Diffusion parameter profiles were created for these two fiber bundles and these profiles were further analyzed with FADTTS. While each of the participants were categorized as 12-month-old for the IBIS study, the interaction between a subject’s actual age in weeks (ranging from 11.8 to 14.5 weeks) and the DTI properties was analyzed to illustrate the use of our tools. On the genu, three areas showed significant interaction between Age and FA, but no areas of significant interaction were found on the splenium (data not shown). **Figure 8C** shows the graphical output of this result from FADTTS in the blue line for Age with the filled in circles indicating significance. **Figure 8E** portrays the same information of significance mapped back on to the original genu fiber bundle.

## DISCUSSION AND CONCLUSION

With the use of diffusion tensor imaging (DTI) on the rise, the field of neuroimaging is in need of a coherent paradigm for localized fiber tract based DTI analysis. Here, we illustrate the UNC-Utah NA-MIC framework for DTI fiber tract analysis assessing the genu and splenium in 1-year-old from the UNC arm of the IBIS study. To demonstrate the use of our framework we analyzed the relationship of subject age in weeks with diffusion tensor properties. For the genu there were three locations of significant interaction as visible in **Figure 8**. The splenium demonstrated no areas of significant interaction. To facilitate utilization of our workflow, all of the data, intermediate files, scripts, and tools are freely available on NITRC.

Over the past few years we have created and tested our tools to provide maximum usability and a detailed analysis of diffusion at each point along fiber bundles. As these are tools for research, the tools in our framework are constantly evolving as we improve their function and incorporate new options. For example, we are currently enhancing DTIPrep to allow for simulation based error estimation, enhancing DTIAtlasBuilder to improve its unbiased atlas building step, as well as providing a GUI (non-Matlab) based interface to FADTTS directly within DTIAtlasFiberAnalyzer.

**FIGURE 9:**
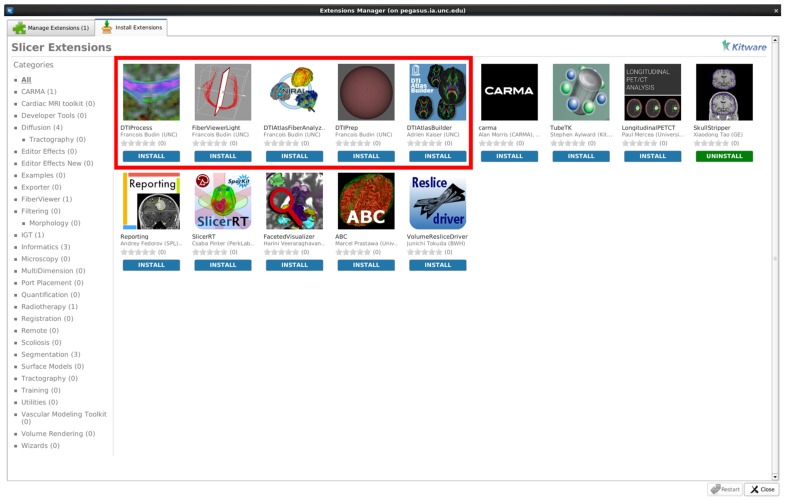
**A screenshot displaying our tools as available extensions in 3D Slicer (box highlighted to indicate our Slicer modules)**.

While all tools employed here are individually available online (see Software and example dataset below), all needed modules of this framework are directly available for download via the 3D Slicer extension manager (version 4.3 and above; **Figure 9**). We hope our workflow will fill the void in the field for the many neuroimaging investigators that need to analyze their DTI data with a localized fiber based approach.

## SOFTWARE AND EXAMPLE DATASET

3D Slicer: 

DTIPrep: 

AutoSeg: 

itk-SNAP: 

DTIAtlasBuilder: 

MriWatcher: 

AtlasWerks: 

DTI-Reg: 

BRAINS: 

ANTS: 

FIberViewerLight: 

DTIAtlasFiberAnalyzer: 

FADTTS: 

Matlab: 

Dataset: 

## Conflict of Interest Statement

The authors declare that the research was conducted in the absence of any commercial or financial relationships that could be construed as a potential conflict of interest.

## References

[B1] Arsigny V. Fillard P. Pennec X. Ayache N. (2006). Log-Euclidean metrics for fast and simple calculus on diffusion tensors. *Magn. Reson. Med.* 56 411–421 10.1002/mrm.20965 16788917

[B2] Avants B. B. Epstein C. L. Grossman M. Gee J. C. (2008). Symmetric diffeomorphic image registration with cross-correlation: evaluating automated labeling of elderly and neurodegenerative brain. *Med. Image Anal.* 12 26–41 10.1016/j.media.2007.06.004 17659998 PMC2276735

[B3] Corouge I. Fletcher P. T. Joshi S. Gilmore J. H. Gerig G. (2005). Fiber tract-oriented statistics for quantitative diffusion tensor MRI analysis. *Med. Image Comput. Comput. Assist. Interv.* 8 131–139 10.1016/j.media.2006.07.003 16685838

[B4] Du G. Lewis M. M. Sen S. Wang J. Shaffer M. L. Styner M. (2012). Imaging nigral pathology and clinical progression in Parkinson’s disease. *Mov. Disord.* 27 1636–1643 10.1002/mds.25182 23008179 PMC3510346

[B5] Faria A. V. Zhang J. Oishi K. Li X. Jiang H. Akhter K. (2010). Atlas-based analysis of neurodevelopment from infancy to adulthood using diffusion tensor imaging and applications for automated abnormality detection. *Neuroimage* 52 415–428 10.1016/j.neuroimage.2010.04.238 20420929 PMC2886186

[B6] Farzinfar M. Oguz I. Smith R. G. Verde A. R. Dietrich C. Gupta A. (2013). Diffusion imaging quality control via entropy of principal direction distribution. *Neuroimage* 82 1–12 10.1016/j.neuroimage.2013.05.022 23684874 PMC3798052

[B7] Geng X. Gouttard S. Sharma A. Gu H. Styner M. Lin W. (2012). Quantitative tract-based white matter development from birth to age 2 years. *Neuroimage* 61 542–557 10.1016/j.neuroimage.2012.03.057 22510254 PMC3358435

[B8] Goodlett C. B. Fletcher P. T. Gilmore J. H. Gerig G. (2009). Group analysis of DTI fiber tract statistics with application to neurodevelopment. *Neuroimage*45(1 Suppl) S133–S142 10.1016/j.neuroimage.2008.10.060 PMC2727755 19059345

[B9] Johnson H. Harris G. Williams K. (2007). *BRAINSFit: mutual information rigid registrations of whole-brain 3D images, using the insight toolkit. Insight J.* Available at:

[B10] Joshi S. Davis B. Jomier M. Gerig G. (2004). Unbiased diffeomorphic atlas construction for computational anatomy. *Neuroimage* 23 S151–S160 10.1016/j.neuroimage.2004.07.068 15501084

[B11] Klein A. Andersson J. Ardekani B. A. Ashburner J. Avants B. Chiang M. C. (2009). Evaluation of 14 nonlinear deformation algorithms applied to human brain MRI registration. *Neuroimage* 46 786–802 10.1016/j.neuroimage.2008.12.037 19195496 PMC2747506

[B12] Lebel C. Gee M. Camicioli R. Wieler M. Martin W. Beaulieu C. (2012). Diffusion tensor imaging of white matter tract evolution over the lifespan. *Neuroimage* 60 340–352 10.1016/j.neuroimage.2011.11.094 22178809

[B13] Liu Z. Wang Y. Gerig G. Gouttard S. Tao R. Fletcher T. (2010). Quality control of diffusion weighted images. *Proc. SPIE* 7628 76280J 10.1117/12.844748 PMC3864968 24353379

[B14] Liu Z. Zhu H. Marks B. L. Katz L. M. Casey B. Goodlett M. S. (2009). “Voxel-wise group analysis of DTI,” in *Proceedings of IEEE International Symposium of Biomedical Imaging* (Boston: IEEE press) 807–810 10.1109/isbi.2009.5193172 PMC3660096 23703686

[B15] Patil R. B. Piyush R. Ramakrishnan S. (2013). Identification of brain white matter regions for diagnosis of Alzheimer using diffusion tensor imaging. *Conf. Proc. IEEE Eng. Med. Biol. Soc.* 2013 6535–6538 10.1109/EMBC.2013.6611052 24111239

[B16] Pieper S. Halle M. Kikinis R. (2004). “3D slicer,” in *1st IEEE International Symposium on Biomedical Imaging: From Nano to Macro* (Arlington: IEEE press) 632–635

[B17] Shi Y. Short S. J. Knickmeyer R. C. Wang J. Coe C. L. Niethammer M. (2013). Diffusion tensor imaging-based characterization of brain neurodevelopment in primates. *Cereb. Cortex* 23 36–48, 10.1093/cercor/bhr372 22275483 PMC3513950

[B18] Smith S. M. Jenkinson M. Johansen-Berg H. Rueckert D. Nichols T. E. MacKay C. E. (2006). Tract-based spatial statistics: voxelwise analysis of multi-subject diffusion data. *Neuroimage* 31 1487–1505 10.1016/j.neuroimage.2006.02.024 16624579

[B19] Song S. K. Sun S. W. Ju W. K. Lin S. J. Cross A. H. Neufeld A. H. (2003). Diffusion tensor imaging detects and differentiates axon and myelin degeneration in mouse optic nerve after retinal ischemia. *Neuroimage* 20 1714–1722 10.1016/j.neuroimage.2003.07.005 14642481

[B20] Van Ginneken B. Heimann T. Styner M. (2007). “3D Segmentation in the clinic: a grand challenge,” in *Proceedings of the workshop on 3D Segmentation in the Clinic at Medical Image Computing and Computer Assisted Interventions* 7–15

[B21] Yushkevich P. A. Piven J. Hazlett H. C. Smith R. G. Ho S. Gee J. C. (2006). User-guided 3D active contour segmentation of anatomical structures: significantly improved efficiency and reliability. *Neuroimage* 31 1116–1128 10.1016/j.neuroimage.2006.01.015 16545965

[B22] Zhu H. Kong L. Li R. Styner M. Gerig G. Lin W. (2011). FADTTS: functional analysis of diffusion tensor tract statistics. *Neuroimage* 56 1412–1425 10.1016/j.neuroimage.2011.01.075 21335092 PMC3085665

[B23] Zhu T. Hu R. Tian W. Ekholm S. Schifitto G. Qiu X. (2013). Spatial regression analysis of diffusion tensor imaging (SPREAD) for longitudinal progression of neurodegenerative disease in individual subjects. *Magn. Reson. Imaging* 31 1657–1667 10.1016/j.mri.2013.07.016 24099667

